# *Solidago virgaurea* L.: A Review of Its Ethnomedicinal Uses, Phytochemistry, and Pharmacological Activities

**DOI:** 10.3390/biom10121619

**Published:** 2020-11-30

**Authors:** Cornelia Fursenco, Tatiana Calalb, Livia Uncu, Mihaela Dinu, Robert Ancuceanu

**Affiliations:** 1 Departament of Pharmacognosy and Pharmaceutical Botany, Faculty of Pharmacy, Nicolae Testemitanu SUMPh, 66 Mălina Mică Street, MD-2025 Chisinau, Moldova; cornelia.fursenco@usmf.md (C.F.); tatiana.calalb@usmf.md (T.C.); 2 Scientific Center of Medicines, Faculty of Pharmacy, Nicolae Testemitanu SUMPh, 66 Mălina Mică Street, MD-2025 Chisinau, Moldova; livia.uncu@usmf.md; 3 Departament of Pharmaceutical and Toxicological Chemistry, Faculty of Pharmacy, Nicolae Testemitanu SUMPh, 66 Mălina Mică Street, MD-2025 Chisinau, Moldova; 4 Department of Pharmaceutical Botany and Cell Biology, Faculty of Pharmacy, Carol Davila University of Medicine and Pharmacy, 6 Traian Vuia Street, Sector 2, 020956 Bucharest, Romania; robert.ancuceanu@umfcd.ro

**Keywords:** *Solidago vigaurea* L., European goldenrod, asteraceae, ethnomedicinal, phytochemistry, distribution, pharmacological activity

## Abstract

*Solidago virgaurea* L. (European goldenrod, Woundwort), Asteraceae, is a familiar medicinal plant in Europe and other parts of the world, widely used and among the most researched species from its genus. The aerial parts of European goldenrod have long been used for urinary tract conditions and as an anti-inflammatory agent in the traditional medicine of different peoples. Its main chemical constituents are flavonoids (mainly derived from quercetin and kaempferol), C6-C1 and C6-C3 compounds, terpenes (mostly from the essential oil), and a large number of saponin molecules (mainly virgaureasaponins and solidagosaponins). Published research on its potential activities is critically reviewed here: antioxidant, anti-inflammatory, analgesic, spasmolitic, antihypertensive, diuretic, antibacterial, antifungal, antiparasite, cytotoxic and antitumor, antimutagenic, antiadipogenic, antidiabetic, cardioprotective, and antisenescence. The evidence concerning its potential benefits is mainly derived from non-clinical studies, some effects are rather modest, whereas others are more promising, but need more confirmation in both non-clinical models and clinical trials.

## 1. Introduction

The genus *Solidago* includes about 190 species and infraspecific taxons (subspecies and varieties) with an accepted status and about 330 species and intraspecific taxons with an ambiguous status [1]. They are widespread throughout the world, most of them originating from North America or confined to this part of the world [2]. Most of *Solidago* species are herbaceous flowering plants, which occur in the spontaneous flora or are cultivated as decorative plants [3]. Raw materials of goldenrods have a long and wide use history in the traditional medicine of different parts of the world: *S. virgaurea* L. (European goldenrod) is the most used in Europe and Asia; *S. canadensis* L. (Canadian goldenrod), *S. gigantea* Aiton (Giant goldenrod), and *S. odora* Aiton—in North America; *S. chilensis* Meyen−in South America [4,5]. According to the Flora Europaea, on the continent, there are 5 representatives of the genus *Solidago*: *S. virgaurea* L., *S. canadensis* L., *S. gigantea* Aiton., *S. altissima* L., and *S. graminifolia* L. Salisb.) [6]. Today, *S. graminifolia* is considered a synonym for *Euthamia graminifolia* (L.) Nutt. [7]. *S. canadensis* and *S. gigantea*, although of North American origin, have become widespread across Europe and are considered “serious invaders”, whereas *S. rugosa*, of the same origins, has been reported only in a few Western European countries [8].

The aerial parts of European goldenrod have been known and used for centuries as anti-inflammatory, spasmolytic, and diuretic remedies in the traditional medicine for the treatment of numerous diseases, especially as a urological agent in kidney and bladder inflammation, urolithiasis, and cystitis [3,4,8,9,10,11,12]. According to the European Medicines Agency, *S. virgaurea* is one of the most used and studied species of the *Solidago* genus in Europe [9].

**Figure 1:**
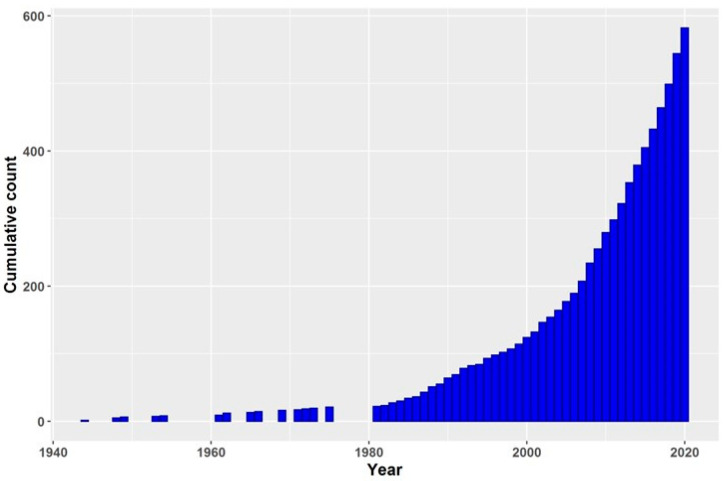
Cumulative number of citations on *S. virgaurea*. Source: PubMed.

The growing interest for the species *S. virgaurea* as a medicinal plant led us to carry out this review using the most relevant and recent international research studies. The scientific community interest in *S. virgaurea* is booming. Figure 1 depicts the cumulative number of articles (total of 580) published on *S. virgaurea* in the period 1944–2020. For this purpose, two well-known and worldwide applied scientific databases (MEDLINE (PubMed) and HINARI), as well as Google Scholar, were used. The number of published paper on this species has escalated in the last two decades (2000–2020).

## 2. General Description, Taxonomy, and Distribution

**Figure 2:**
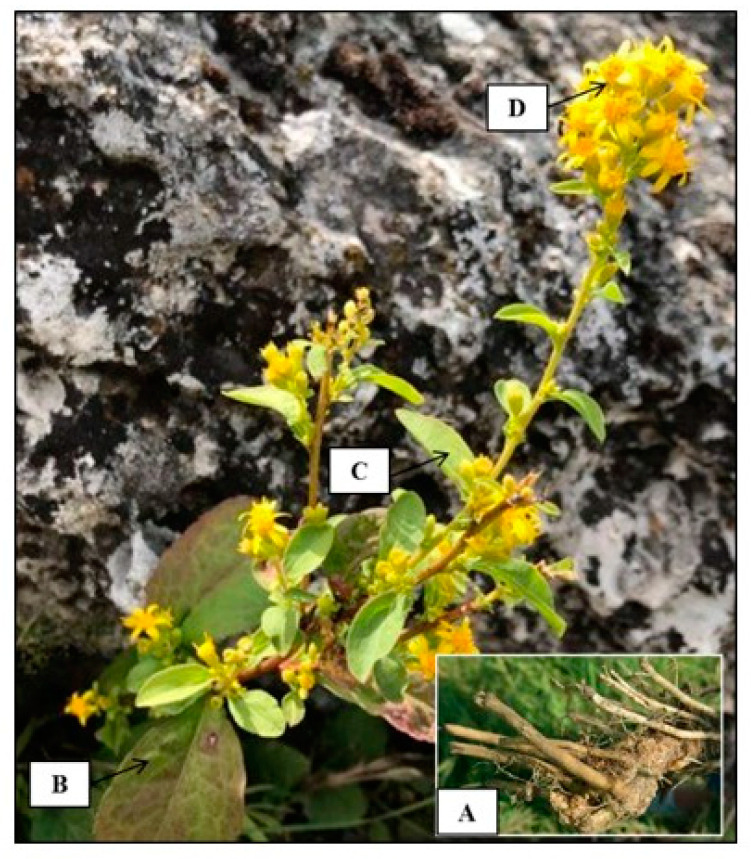
*S. virgaurea* (original photo): (**A**) oblique rhizome, (**B**) basal leaves, (**C**) cauline leaves, (**D**) radiate flower heads.

*S. virgaurea* is a perennial herb provided with an oblique, woody rhizome, of a cylindrical shape and devoid of knots, on which stem scars are visible (Figure 2A). The round, erect stem may achieve a height of up to 1 m, and is ramified and pubescent at the topside. The leaves, with an alternate arrangement, are simple, slightly pubescent on the adaxial face, and pubescent on the abaxial one. Basal leaves have ovate or ovate-elliptic blades with an acute tip and a winged petiole, whereas upper leaves have short stalks and linear-lanceolate or elliptic blades, with margins either serrated or entire (Figure 2B,C). The radiate flower heads have morphologically distinguished ray (female, tongue-like) and disc yellow florets (hermaphrodite, tubular). The flowers capitula are grouped in a simple raceme or in panicles (Figure 2D). The receptacle is glossy and flat. The fruit is a cylindrically-shaped achene, with 8–10 ribs and a pappus derived from the modified calyx [13].

A recent morpho-anatomical local study focused on the morphological and anatomical investigation of *S. virgaurea* from flora of the Republic of Moldova, and its herbal product *Solidaginis virgaureae herba,* was done by Calalb T. et al. [13]. The anatomical features of the highest interest for the exact identification of *S. virgaurea,* established in this study, include: repartition of stomata on both sides of the leaf, anomocytic arrangement of stomata, cone-shaped or fan-shaped multicellular trichomes on both epidermises, as well as glandular trichomes, dorsiventral structure of the leaf with vascular bundles collateral and open, and secretory ducts in the stem. The anomocytic stomata reported for the leaf is consistent with the findings of Szymura M. and Wolsky K. [14], who also established that anomotetracytic stomata were the most widespread type in other *Solidago* taxa collected from Poland. The presence of two categories of multicellular trichomes (cone-shaped and fan-shaped) on the goldenrod leaf was also reported by other sources [14,15]: by Buynov MV. on *S. dahurica* leaf [16], by Douglas M. et al. on *S. chilensis* leaf [17], and by Fedotova VV. on *S. caucasica* leaf [18]. Care is needed for a correct identification, because in the 19th century, a scientific paper written by a medical doctor (based on his personal experience repeated several times) drew the attention to the risks of confusing this medicinal plant with a non-effective (and likely dangerous for the health) *Senecio nemorensis* L. [19]

According to a number of sources [20,21], *S. virgaurea* is regarded as a taxonomic group or complex, and it consists of perennial herbaceous species extensively distributed from Europe to East Asia. As a group, it is generally divided longitudinally: in Europe the genus is represented by *S. virgaurea* L., in Siberia and most of the Far East by *S. dahurica* (Kitag.) Kitag. ex Juz. Together with *S. spiraeifolia* Fisch. ex Herder, whereas in the Far East region of Russia, known as Chukotka and in North America, the genus is represented by *S. multiradiata* Ait. [22].

The European *S. virgaurea* L. has been described as “an exceedingly polymorphic taxon”, and a multitude of narrowly related taxa have been included within it at different levels (varieties, subspecies, and even species) [22,23]. In a European country (Czech Republic) flora, discussing the variability, S. Slavik (2004) has identified 17 taxa as belonging to the *S. virgaurea* L. group (leaving aside taxa from Japan), of which a number of six are qualified as subspecies, whereas a number of eleven as “microspecies” [22,24]). The Atlas of the British and Irish flora [25] describes *S. virgaurea* as highly variable, with many distinct forms for distinct habitats (ecotypes). Strong correlations have been claimed between *S. virgaurea* genotypes and their geography, and a strong ability to rapidly evolve and ecologically diversify has been recognized for the species [26].

## 3. Synonyms and Common Names

According to the Assessment Report on *Solidago virgaurea*, realized by the European Medicines Agency [9] and the World Flora Online [1], the main synonyms in use are: *Amphiraphis leiocarpa* Benth., *Amphiraphis pubescens* DC., *Aster virgaurea* (L.) Kuntze, *Dectis decurrens* Rafin. (Lour.), and *Doria virgaurea* Scop. *S. patagonica* Phil. is currently an accepted name [1], although it was reported that one Argentine specimen identified as *S. patagonica* was in fact an escaped cultivar of S. virgaurea [27].

The most often used common name for *S. virgaurea*, as well as for other *Solidago* species is goldenrod. Sometimes the “European” qualifier is added to this vernacular name and in this paper, in order to avoid confusion with other species of the genus, we will hereafter use this name (European goldenrod). According to the American Botanical Council, other common names in use are: Solidago, Virgaurea, Woundwort, Aaron’s rod, and Yellowweed [28]. The dried flowering aboveground parts are the subject of a European herbal monograph [29].

## 4. Ethnomedicinal Uses

*S. virgaurea* has a diversity of medicinal uses in the territories where it is spread. Probably its most widely known ethnopharmacological uses are related to kidney disorders (being often found in teas intended to help pass kidney calculi), urinary tract infections, the overactive bladder syndrome, and prostatic diseases [30,31,32], the urologic uses of the plant going back at least to the writings of Arnold von Villanova (1240–1311) [33]. Traditionally, the aerial parts of the plant have been used for healing and antiseptic properties [9], as well as for the treatment of diabetes, allergies, and gastro-intestinal disorders [8,32]. Likewise, infusions or decoctions prepared from European goldenrod is used in the traditional medicine in many parts of the world for its antibacterial and anti-inflammatory effects [34], including inflammation of the oral cavity and throat, when used as a mouth rinse [32]. Further scientific studies have shown the growing importance of European goldenrod as a source for herbal drugs [35]. In many European countries, the herbal product derived from the species has often been in combination products [9].

### 4.1. Germany

Hieronymus Bock (1498–1554), one of the first modern botanists in Germany, conjectured that Germanic tribes had been using the plant for medicinal purposes, mentioning that they regarded it as a “miracle herb” (*Wunderkraut*) [36]. It is believed that the German father of Reformation, Martin Luther (1438-1546), had a good opinion on goldenrod and used it often to care for his physical infirmities [33]. One of the first reports on its diuretic and anti-inflammatory effects are ascribed to the “father of German botany“ (1525–1590), Jacobus Theodorus Tabernaemontanus [37], who stated that it “also cleanses the kidneys and urinary tract of all coarse mucus” [36]. The name *Heydnisch Wundkraut* (heathen woundwort), employed in the German territories during the Middle Ages for the plant, evokes the healing properties of the herb. Another vernacular German name, *Unsegenkraut* (curse herb), indicates belief at that time in its magic abilities, in an era where disease was often attributed to witchcraft and metaphysical causes, and indirectly the name might still point towards its potential medicinal properties [38]. In German folk medicine, goldenrod was used for the treatment of urinary retention, kidney stones, and hemorrhoids [36]. Since the middle 19th century, its use was slowly forgotten in Germany, to be revived only relatively recently with the renewed interest for the herbal therapy [36].

Currently, a well-established use is accepted in Germany for inflammatory diseases of the urinary tract collection system, urolithiasis and renal gravel. A variety of extracts are used, particularly dried extracts obtained from the aerial parts (*Solidaginis virgaureae herba*) using 30–60% ethanol as an extraction solvent [9]. A monograph of the species was introduced in DAB in 2002, which also acknowledged *S. canadensis* and *S. gigantea* as valid species, despite certain differences in the spectrum of their phytochemicals [33].

### 4.2. Czech Republic

The herbal drug obtained from *S. virgaurea* is included in the Czech Pharmacopoeia 2009 [39]. A drink obtained from the aerial parts of the plant is used as an adjuvant treatment in inflammatory conditions of the urinary system, as well as for the prevention of kidney and bladder calculi [9]. It is not clear, though, whether such uses represent an old Czech tradition, as a Czech paper on the species only cited foreign sources when referring to the traditional medicinal use of the species [39].

### 4.3. Poland

Traditionally, the infusion prepared of dried aerial parts of *S. virgaurea* has been used as a diuretic and as an adjuvant in treatment of minor complaints of the urinary tract [9]. In a Polish source, it is stated that raw material of this herb is characterized by diuretic, detoxifying, anti-inflammatory, and bile secretion enhancing properties [35]. A Polish source mentions its disinfectant properties as the most important among the traditional uses, but also its useful effects in accelerating wound healing and in skin care [40].

### 4.4. Russian Federation

In Russian folk medicine, European goldenrod is used for a variety of conditions, from gallstone disease to indigestion, and from rheumatism to gout. For external use, fresh leaves are recommended in abscesses and boils [41]. Other Russian sources [35,42] state that most common uses of this species include prevention and treatment of various diseases of the kidneys, bladder, and prostate gland (i.e., the traditional use most widely acknowledged in Europe). In the Russian folk tradition, the European goldenrod is also known as a hemostatic and astringent agent, as well as a good remedy for respiratory diseases (tonsillitis, laryngitis, acute respiratory diseases), gallstone diseases, and pulmonary tuberculosis [41].

### 4.5. Ukraine

The use in tuberculosis is also well established in the Ukraine folk medicine, where the name of the plant, *zolotushnik*, alludes to its use in decoctions as “a good remedy against scrofula (*zolotukha*)”, but the plant was also believed to have diuretic effects [43].

### 4.6. Bulgaria

According to a Bulgarian source, the aerial parts of *S. vigaurea* L. are used as a diuretic, antihypertensive, and expectorant, as well as in the therapy of renal deficiency and gout [44].

### 4.7. Romania and the Republic of Moldova

*S. virgaurea* has a long history of use in the Romanian traditional phytotherapy. The herbal product *Solidaginis virgaureae herba* has been used in herbal therapy and marketed by specialized outlets since 1990 [45]. Ethnopharmacological uses of this plant are mostly related to maintaining the health of the urinary tract and the normal functioning of the digestive system. Traditionally, it is recommended as a diuretic, saluretic, anti-inflammatory, antiseptic, healing, or a sedative agent. For external use, the most common application consists in administering an infusion or decoction of the aerial parts or blooming tops in the treatment of wounds or ulcers of the oral cavity [46,47]. The external use in rickets, also acknowledged by Romanian traditional sources [48], does not seem to have great benefit, considering the current knowledge of this disease and its causes.

### 4.8. Korea

The root and aerial parts of *S. virgaurea* subsp. *gigantea* (Nakai) Kitam have been used as an appetite stimulant and diuretic in Korean folk medicine, whereas the immature aerial parts are used in the same area as food [49,50].

### 4.9. China

Decoctions obtained from the whole plant were used for their antibacterial activity, and in respiratory tract infections for its expectorant and anti-inflammatory properties [51].

### 4.10. Other Uses

Besides its medicinal uses, *S. virgaurea* has been recognized as a first-class alternative source for the floriculture business, both on the old continent and in the new world [32]. It has also been proposed to be used as a rotation crop as a means of containing noxious weeds in an organic agriculture context [35]. *Solidago* spp., including *S. virgaurea*, have been claimed to have potential utility for phytoremediation purposes, based on their ability to transfer iron from soil to plants near iron processing industrial sites [35]; however, studies on other oligo-elements (such as zinc) have not identified particular hyperaccumulator properties for the plant [52], and whereas a number of papers have been published on the phytoremediation potential of *S. canadensis* [53,54], we could find none on *S. virgaurea*. The pollen of the latter is of good quality, and its availability may contribute to long-lived bees being able to survive a hard winter [55].

## 5. Phytochemistry

Extracts of *S. virgaurea* contain C6-C1 glycosides (virgaureoside, leiocarposide) and aglycones (vanillic acid, gallic acid) [4,9,56,57,58], C6-C3 polyphenolic acids (caffeic, chlorogenic, ferulic, synapic, 3-hydroxyphenylacetic acid, 3,4-dihydroxyphenylacetic, homovanilic, acids) [3,9,10,45,53,54,55,56,57], a number of flavonoid molecules (mostly quercetin and kaempferol glycosides, as well as the free aglycons and small amounts of cyanidin derivatives) [3,4,9,45,49,59,60,61,62,63,64,65], oleanane-type triterpene saponins [9,66,67,68,69,70,71,72,73], essential oils containing monoterpenes (alpha-and beta-pinene, myrcene, limonene, sabinene) [35,74,75,76,77] and sesquiterpenes (germacrene D β-caryophyllene, α-humulene,), clerodane-type diterpenes [78], polysaccharides [79], and polyacetylenes [80] (Table 1). The European Pharmacopoeia monograph for *Solidaginis virgaureae herba* regards flavonoids as quality markers and for the product requires a content of at least 0.5% and maximum 1.5%, expressed as hyperoside [81].

**Table 1:** Chemical compounds identified in *S. virgaurea.*

Chemical Compounds	Place/Country of Collection	References
	Flavonoids (Figure 3)	
Quercetin, Quercetin-3-*O*-glucoside (isoquercitrin) Quercetin-3-*O*-galactoside (hyperoside) Quercetin-3-*O*-rhamnoside (quercitrin) Quercetin-3-*O*-rutinoside (rutin) Quercetin-3-*O*-arabinopyranoside (avicularin) Kaempferol-3-*O*-glucoside (astragalin) Kaempferol-3-*O*-rhamnoside (afzelin) Kaempferol-3-*O*-rutinoside (nicotiflorin) Kaempferol-3-*O*-robinobioside (biorobin) Myricetin 3-rhamnoside (myricitrin) Isorhamnetin-3-*O*-rutinoside (narcissin) Cyanidin-3-gentiobioside mono-C-glycosylflavones (?) di-C-glycosylflavones (?)	Poland, Italy, Hungary, Korea, Romania, Lithuania	Fuchs L. [82], Budzianowski J. et al. [61], Borkowski B. and Skrzypczakowa L. [60], Chodera A. et al. [65], Roslon W. et al. [59], Pietta P. et al. [62], Apáti P. et al. [83], Choi SZ. et al. [49], Tamas M. [63], Dobjanschi L. et al. [45,64], Kraujaliene V. et al. [84]
**C6-C1 Compounds (Figure 4)**		
Benzoic acid 3-Hydroxybenzoic acid 4-Hydroxybenzoic acid 3,4-Dihydroxybenzoic (protocatechuic) acid Salicylic acid Gentisic acid Vanillic acid Gallic acid Leiocarposide 2-methoxybenzyl-2,6-dimethoxybenzoate	Poland Egypt Korea	Kalemba D. [85], Abdel Motaal A. et al. [10], Choi SZ. et al. [49], Thiem, B. et al. [58], Bajkacz S. et al. [86], Sung JH et al. [50],
**C6-C2 and C6-C3 Compounds (Figure 5)**		
Caffeic acid, Chlorogenic acid 5-*O*-caffeoylquinic (neochlorogenic) acid 3,5-di-*O*-caffeoylquinic acid 3,4-di-*O*-caffeoylquinic acid 4,5-di-*O*-caffeoylquinic acid 3,4,5-tri-*O*-caffeoylquinic acid Methyl 3,5-di-*O*-caffeoylquinate 3-hydroxyphenylacetic acid 3,4-dihydroxyphenylacetic acid 5-*p*-Coumaroylquinic acid Homovanilic acid *p*-Coumaric acid Ferulic acid Sinapic acid Rosmarinic acid	Poland Egypt Korea Iran	Kalemba D. [85], Abdel Motaal A. et al. [10], Choi SZ. et al. [49], Thiem B. et al. [58], Bajkacz S. et al. [86], Haghi G., Hatami A. [87], Roslon W. et al. [59], Kraujalienė V et al. [84], M. Marksa et al. [88], D. Fraisse et al. [89], Borkowski B. and Skrzypczakowa L. [60], Jaiswal R. et al. [90]
	**Coumarins**	
7-hydroxy-coumarin (umbelliferone)	Czech Republic	Dobias P. et al. [91]
**Terpene Derivatives (Figure 6)**		
α-Pinene, β-Pinene, Sabinene Myrcene Limonene β-Ocimene Germacrene-D, β-Caryophyllene, α-Humulene, Clerodane diterpenes 2,8-(*cis*)-(*cis*)-Matricaria ester Matricaria γ-lactones Lachnophyllum lactone ent-germacra-4(15),5,10(14)-trien-1β-ol β-dictyopterol	Poland, Japan, Italy, Russian Federation, USA Denmark Korea	Kalemba D. [74], Kalemba D. and Thiem B. [75], Fujita S. [76], Bertoli A. et al. [77], Tkachev AV. et al. [35], Goswami A et al. [92], Starks CM. et al. [78], Lam J. [80], Choi S. [49]
**Saponins (Figure 7)**		
Virgaureasaponins 1–6 Solidagosaponins X-XXIX Bellisaponin BA2 Erythrodiol-3-acetate	Germany, France, Romania Japan	Bader G. et al. [34,56,57,58,59], Chevalier M. et al. [86], Laurençon L. et al. [61], Dobjanschi L. et al. [85], Inose Y. et al. [60], Sung JH et al. [50]
	**Carbohydrates and Other Compounds**	
Polysaccharides α-tocopherol quinone 2-phyten-1-ol	Russian Federation Korea	Pychenkova PA. [66], Sung JH et al. [50]

It has been speculated [3] that a number of the active compounds of *S. virgaurea* extracts (leiocarposide, polyphenolic acids, flavonoids, saponins) exert a synergistic activity in displaying the reported anti-inflammatory effects of the product [56,93]. The antioxidant activity has been attributed to the polyphenolic compounds [3,94,95,96], while flavonoids are thought to be responsible for the spasmolytic effects [3,31,97].

### 5.1. Flavonoids

Among the flavonoids, rutin, quercetrin, astragalin, nicotiflorin, biorobin, and narcissin have been considered “the most representative” [62], and they are accompanied by their aglycons [49,84]. More recently, flavanones aglycones and glycosides have also been detected and quantified in the different parts of the plant: eriodictyol (the largest amount in the flowers, followed by leaves and then stems, mostly as glycosides), naringenin (similar quantitative distribution in flowers, leaves, and stems, mostly as glycosides), and very small amounts of hesperitin (not assessed separately in each aerial parts) [98]. Eriodictyol and naringenin are present in the form of both R and S enantiomers, whereas hesperitin was detected only as the S enantiomer [98]. All flavonoid heterosides seem to be 3-*O*-glycosides, as for the majority of *Solidago* species (the notable exception being *S. graminifolia* (L.) Salisb, in which mono- and di-C-glycosylflavonoids have also been reported [58], but which now is considered a synonym for the *Euthamia graminifolia* (L.) Nutt [1]). The presence of flavonoid-C-heterosides in *S. virgaurea* has also been occasionally claimed in secondary sources [59], but we could not locate a primary reference reporting them. Cyanidin-3-gentiobioside is the main anthocyanin present in the leaves, but at least one other cyaniding-glycosyde was reported in very small amounts [99]. Flavonoid glycosides tend to be better extracted in ethanol of 70% or higher concentrations [83]. As mentioned above, hyperoside is considered the key flavonoid by the European Pharmacopoeia [81]. However, in one paper, quercitrin was the major phytochemical from a quantitative standpoint [84], whereas other sources reported rutin as the dominant flavonoid [59,60] (a mean content 196.42 mg 100 g^−1^ reported by [59]). A qualitative and quantitative comparative study of flavonoids from extracts of four *Solidago* spp. reported in Romanian flora was carried out by Dobjanschi L. et al. [38,49], who found a total flavonoid content for *S. virgaurea* of 4.06%, expressed as rutin. They also found that for *S. virgaurea*, specific was the presence of rutin and hyperoside, whereas quercitrin (unlike the findings of V. Kraujalienė et al.) was absent [45,64]. Spasmolytic effects (as discussed below) [31,97], diuretic activity [10], have been attributed to the flavonoids, and other effects have also been specifically ascribed (at least partially) to some flavonoids, e.g., antiadipogenic effects to kaempferol-3-*O*-rutinoside [100].

### 5.2. C6-C1 Compounds

Several main compounds with a C6–C1 have apparently been reported up to date in *S. virgaurea*, two glycosides and two aglycones: virgaureoside A, a bis-desmosidic glycoside derived from benzoic acid (2-beta-D-glucopyranosyloxybenzoic acid-2′-beta-D-glucopyranosyloxybenzyl ester) [101], leiocarposide (2′-hydroxybenzyl-3-methoxybenzoate 2′,4-diglucoside) [56], vanillic acid, gallic acid [9,102], benzoic acid, 3-hydroxybenzoic acid, 4-hydroxybenzoic acid, 3,4-dihydroxybenzoic (protocatechuic) acid, and 2,5-dihydroxibenzoic (gentisic) acid [85,86]. Such derivatives of the benzoic acid are often occurring in the family Asteraceae [103].

A somewhat detailed history of the discovery of the two phenolic glycosides was provided by L. Skrzypczak et al. [103]. Leiocarposide is currently considered the most important and was found to be maximally biosynthesized in the flower buds (1.60%) and in the two-year leaves, post-blooming (1.05%) [104]. Similar contents (0.4–1.6%) were reported by other researchers, depending on a number of variables such as plant height at harvest, collection time, or the natural state of the herbal samples [58]. Leiocarposide has attracted interest for its pharmacological potential, being explored for its hypothesized anti-inflammatory, analgesic, antilithiatic, and diuretic effects, as discussed below. For plants cultivated in vitro, the content of leiocarposide is lower than that of naturally growing plants (0.18% vs. 0.2–1.0%) [58].

### 5.3. C6-C2 and C6-C3 Compounds

A variety of C6–C3 phenolic acids have been identified in different studies. Among the most important are the caffeoylquinic derivatives (chlorogenic acid, but also 5-*O*-caffeoylquinic (neochlorogenic) acid, 5-p-coumaroylquinic acid, 3,5-di-*O*-caffeoylquinic, 3,4-di-*O*-caffeoylquinic, 4,5-di-*O*-caffeoylquinic acids, methyl 3,5-di-*O*-caffeoylquinate, and 3,4,5-tri-*O*-caffeoylquinic acid, the latter showing superior anti-inflammatory effects over the di-caffeoylquinic derivatives.) [10,49,58,59,86,90]. Caffeic, p-coumaric, ferulic, sinapic, 3-hydroxyphenylacetic, 3,4-dihydroxyphenylacetic (DOPAC), and homovanilic acids were also reported in phytochemical studies of *S. virgaurea* L. [86]. In one study, whereas chlorogenic acid was detected and measured (by HPLC), ellagic and rosmarinic acids could not be detected [87]. Chlorogenic acid content may vary considerably, as shown in one study that measured it in samples collected from 20 sites, which found concentrations as low as 158.99 mg/100 g and as high as 441.50 mg/kg [59]. Similarly, rosmarinic acid varied in the same study between 256.38 mg/100 g and 898.70 mg/100 g [59], whereas (as mentioned) in a different study, it could not be detected at all [87]. As already discussed in the literature [88], a number of variables, such as the herbal part, ontogenetic development stage, and ambient conditions have a considerable influence on the qualitative and quantitative content in phenolic compounds of the species.

### 5.4. Coumarins

Up to date, a single paper [91] reported the presence of a coumarine compound in *Solidago virgaurea*: umbeliferone (7-hydroxy-coumarin). Esculetin and scopoletin were not detected in the species [91].

### 5.5. Terpene Derivatives

A number of over 60 compounds have been described in the essential oil obtained from the flowering tops of *S. virgaurea* L. collected in Poland [74], whereas in specimens from Lithuania, 106 compounds were identified in flowers and 95 in leaves [105]. The key compounds (as found in three samples from three different sites of Poland) were the monoterpenes α-pinene (27.4–34.1%), myrcene (7.8–17.9%), β-pinene (5.4–7.5%), limonene (3.0–14.1%), and sabinene (0.4–11.8%), as well as the sesquiterpenes germacrene D (8.2–17.0%), and in smaller amounts α–humulene, β-caryophyllene, and α–muurolene [74]. In Lithuanin specimens, the composition reported recently was rather different: in the leaves, the most important compounds detected were caryophyllene oxide, trans-verbenol, spathulenol, humulene epoxide II, α –pinene (only 5.21%), and germacrene D; in flowers of the same origin, the most important compounds were caryophyllene oxide, humulene epoxide II, germacrene D, trans-verbenol, spathulenol, and bornyl acetate [105]. Detailed information on the chemical constituents of the essential oil is presented in Table 2.

Looking at all compounds grouped by chemical structure, the largest proportion in the Polish samples consisted of monoterpene hydrocarbons (58–73%) and sesquiterpene hydrocarbons (17–31%); oxygenated monoterpenes and sesquiterpenes (about 3% each), benzoic acid and salicilyc acids (about 1%) represent less than 10% of the total oil [74]. The essential oil of specimens cultivated in vitro by micropropagation was similar in its monoterpene contents, but the sesquiterpenes were apparently less represented for *S. virgaurea* L. in this case [75]. A.V. Tkachev et al. (2006) compared the composition of the essential oil from the aerial parts harvested from two different heights in the Russian Altai and found that the product harvested at a lower height (290 m) are richer in essential oil (0.22%), which contains higher amounts of α-pinene and myrcene as compared to the oil (0.07%) obtained from plants harvested at a more elevated height (650 m) [35]. The key compounds in this study were almost the same as in the specimens from Poland, the only notable exception being a higher content in β-caryophyllene for both specimens (maximum content in the Polish specimens was 3.3%, whereas in the Russian specimens the minimum content was 6.3%) [35,74].

Besides the essential oil terpenes, from the aerial parts of *S. virgaurea* L., a number of at least 12 *cis*-clerodane lactones were reported in the 1980s, eight of which were new at the time of reporting [92]. In 2010, an additional set of nine new clerodanes were isolated from the species (most likely the aerial part, although up to date, many clerodanes have been isolated from roots of different *Solidago* species), seven of which have the relatively atypical feature of a carboxylic acid at C-19 (solidagoic acids C-I) [78].

A number of polyacetylene compounds have been reported in the roots of the species (matricaria ester, two matricaria-γ-lactones, one lachnophyllum lactone), that show considerable seasonal variation [80]. Only the matricaria ester could be detected in the aboveground shoots, whereas the other compounds are absent from the aerial parts [80].

### 5.6. Saponins

The saponins of *S. virgaurea* L. have been an object of study as early as the 1930s, but the first data on their structure were provided by K. Hiller et al. in 1975 [106]. In the 1980s, G. Bader et al. were among the first to isolate and establish the chemical structures of deacylated saponins found in the aerial parts, named by the authors virgaureasponins 1–3 [37,66,68,107]. Around the same time, in Japan, Y. Inose et al. isolated and elucidated, from samples of Japanese origin, the structures of oleanane-type solidagosaponins I-XX, many of them glycosylated in position 16 of the aglycone alone or besides position 3 [51,70], and later the same group further identified solidagosaponins XXI-XXIX [108]. The same Japanese researchers reported the presence of bellisaponin BA2 [108], which had been isolated previously from *Bellis perennis* L. [109]. Later, another European group isolated virgaureasaponins 4–6 from *S. virgaurea* ssp. *alpestris* [73]. Quantitatively, the saponin content is similar between *S. virgaurea*, *S. gigantean*, and *S. canadensis* [71]. Unlike *S. canadensis* and *S. gigantea*, which are derived from byogenin and have more complex sugar chains, the saponins isolated from *S. virgaurea* are derived from polygalacic acid and are acylated by carboxylic acids [37]. As for many phytochemicals, some of the saponins have multiple synonyms, sometimes confusing; for instance, solidagosaponin XVIII is also known as virgaureasaponin C [73].

### 5.7. Polysaccharides

Both inflorescences and leaves of *S. virgaurea* L. contain polysaccharides. Those isolated from inflorescences contain uronic acids (over 40%, mostly galacturonic acid), galactose (13–18%), glucose (7–12.5%), rhamnose (4–7.5%), arabinose (2–8%), and xylose (1–2%) residues [79]. The amount and the proportion among the components vary along the growing stages of the plant [79]. J. Saluk-Juszczak (2010) reported quite different proportions of sugars for the polyphenolic-polysaccharide conjugates isolated from the flower heads (rhamnose 22.4%, fucose 5.0%, arabinose 19.2%, xylose 2.6%, mannose 1.3%, glucose 13.0%, galactose 14.0%) [110].

## 6. Pharmacology

### 6.1. Antioxidant Properties

Joining the nutritional vocabulary of the masses at least three decades ago, the concept of “antioxidants” remains one poorly understood in the field of life sciences, and the clinical relevance of antioxidants is still rather fuzzy, with many knowledge gaps [111]. However, partly because the assessment of antioxidant potential is easily accessible and relatively cheap, for many herbal products, the antioxidant properties are evaluated repeatedly, and *S. virgaurea* makes no exception, the first study on this topic dating from 1995 [96]. In this study, ethanolic extracts of the plant were shown in vitro to inhibit lipoxygenase and xanthine oxidase pathways [96]. A methanol extract obtained from the young shoots and leaves had stronger antioxidant effects (measured in vitro with a DPPH-based assay) than an extract prepared with hot water [95]. Autoclaving of an 80% ethanol extract resulted in decreased scavenging effects on DPPH and ABTS (associated with a decline in polyphenol and flavonoid contents), but it increased the chelating effects on ferrous ions [112]. Although a slightly higher antioxidant effect was observed when using pressurized fluid extraction over the ultrasonic extraction method on *S. virgaurea* L. leaves (94.0% vs. 89.0% inhibition), the difference was not statistically significant [91]. Both leaf and stem powders, as well as extracts obtained from those parts, prevented lipid oxidation when applied on ground pork samples [113,114]. Among 23 herbal species examined for their antioxidant effect in one study, *S. vigaurea* L. had an average antioxidant activity (in decreasing order, it occupied the 11th position out of 23) [44].

The key component and marker of the antioxidant properties of *S. virgaurea* was found by M. Marksa et al. (2020) to be 3,5-dicaffeoylquinic acid (about half of the whole scavenging activity, as for *S. canadensis* and *S. ×niederederi*, but unlike *S. gigantea*, for which the main component responsible is chlorogenic acid) [88]. For several *Solidago* species tested, the scavenging activities were stronger for the leaf products than for the inflorescences [88]. The radical neutralizing properties of quercetin derivatives from *Solidago* species was shown to be considerably lower to that of the cafeoylquinic derivatives, but it is higher than that of kaempferol derivatives, whose antioxidant potential is insignificant [88]. Di-caffeoylquinic acids have a stronger radical scavenging effect than mono-caffeoylquinic acids [88].

### 6.2. Anti-Inflammatory Effects

The anti-inflammatory activity of *S. virgaurea* extracts or components isolated from the species has been repeatedly evaluated, confirmed, and ascribed to different phytochemicals from its composition. In a rat model, H. J. Jacker et al. (1981) showed that a triterpene saponin fraction administered i.v. at a low dose (1.25–2.5 mg/kg) caused a significant decrease of edema, as measured by pletysmograph [9,115]. Although J. Metzner et al. (1984) reported some anti-inflammatory effect for leiocarposide (200 mg/kg) in a carrageenan-induced edema model in rats, the effect was obviously inferior to that of phenylbutazone (e.g., 3% vs. 53% two hours post-administration and 27% vs. 54% reduction five hours post-administration) [9,56]. Rutin and quercetin, as well as 3,5-dicaffeoylquinic acid were shown by M. Melzig et al. (2000) to inhibit leukocyte elastase, an effect considered synergistic with the radical scavenging of the same molecules in exerting their anti-inflammatory activity [33,93]. Instead, saponins and leiocarposide did not demonstrate any such elastase inhibition; the same authors claimed that ester saponins stimulate the release of ACTH by their interaction with cell membranes of the pituitary cells, and thus, consecutively, glucocorticoids with anti-inflammatory effects [33,93]. 3,4,5-*O*-tricaffeoylquinic acid was identified among phenolic compounds from *S. virgaurea* as having the highest anti-inflammatory effect (88% of that of indomethacin) in rats (carrageenan-based rat paw edema) and to inhibit TNF-α and IL-1β [10]. On the other hand, other studies reported stimulation of TNF-α secretion by macrophages, an effect induced by different phytochemicals of the species (2-methoxybenzyl-2-hydroxybenzoate, benzyl-2-hydroxy-6-methoxybenzoate) [116], and it remains to be investigated in what contexts and under the influence of what variables one or the other effect will predominate.

Aqueous and ethanolic extracts of *S. virguarea* have demonstrated an ability to reduce paw edema and arthritic paw volume in rat models of inflammation [117]. Some inhibition of dihydrofolate reductase has been described for a hydroalcoholic extract of the species, and this has been suggested as contributing to the anti-inflammatory effects of the *S. virgaurea* extracts, being known that inhibitors of the enzyme, such as methotrexate, do have anti-inflammatory activity [118,119,120].

A standardized combination of alcoholic extracts of *S. vigaurea*, *Populus tremula* L., and *Fraxinus excelsior* L. has been developed as an anti-rheumatic drug and has been relatively extensively investigated [117,121,122].

### 6.3. Analgesic Activity

An in vitro study evaluated the analgesic potential of a methanol seed extract by assessing its affinity for three receptors involved in acute pain signaling (bradykinin, neurokinin 1, and calcitonin gene related peptide). The extract exhibited substantial binding to the bradykinin receptor, but this effect was canceled by PVP treatment, allowing the authors to speculate that it should probably be ascribed to non-specific binding of tannins or other polyphenols [123]. In the hot plate test on mice, leiocarposide demonstrated very similar analgesic effects to aminophenazone for the first hour, but those effects almost disappeared post-administration after the second hour [56].

### 6.4. Spasmolytic and Antihypertensive Activity

Ex vivo data obtained on isolated smooth muscles from guinea pig gut showed a modest spasmolytic effect for a *S. virgaurea* ethanol extract (less than 15% of the papaverine effect) [9,124]. As mentioned below, extracts of *S. virgaurea* have demonstrated anti-muscarinic effects on isolated bladder, inhibiting the M2 and M3 receptors [31]. It has been stated that all species of the genus have “hypotensive activity”, including *S. virgaurea*, which demonstrated such an effect in dogs, for a leaf extract, at a dose of 150/kg [119,125,126]. Aqueous extracts from flowers and from leaves, administered by i.v. route in rats, at doses of 180 and 360 mg/kg found no reduction of blood pressure after the first 2–5 min, for both extracts [127], despite isolated interpretations to the contrary [119]. This has been speculatively related to a potential contribution of flavonoids, based on the reported vasodilatory effects mediated by the inhibition of protein kinase C resulting in the relaxation of the arterial smooth muscle [97]. In that study, flavonols had a stronger effect than flavones, which in their turn had a more potent effect than flavanols [97], and as shown above, *S. virgaurea* key flavonoids are derivatives of flavonols (quercetin and kaempferol).

### 6.5. Diuretic Effects and Benefits in Other Urinary Tract Conditions

As shown above, many folk traditions attribute a diuretic activity to extracts prepared from *S. virgaurea* L. In academic sources, it is mentioned at least starting with the 17th century, when Shcroeder’s “Thesaurus pharmacologicus” points out to it; throughout the 20th century, different sources also cite this pharmacological effect in relationship to *S. virgaurea* products [9].

The diuretic effects have been attributed to the flavonoid fraction (particularly quercetin and its derivatives), which was shown to inhibit the neutral endopeptidase, resulting in an enhanced urinary flow [88,93]. The inhibition of neutral endopeptidase leads to an increase in the plasma concentration of natriuretic peptides, which have strong natriuretic properties [128]. A. Chodera et al. (1991) found that this flavonoid fraction (25 mg/kg b.w.) increases the urine output in rats by about 88%, and causes a decrease in the excretion of sodium and potassium, accompanied by an increase in calcium excretion [9,65]. These results are in contradiction with those reported later by U. Kaspers et al. (1998), who found no increase in urine volume or electrolytes for the flavonoid fraction [9,129]. Instead, they reported that the hydroxycinnamic acid fraction (100 mg/kg) and the saponin fraction (25–100 mg/kg) had an effect comparable with those of furosemide [9,129], including an increase in sodium and potassium excretion, unlike the data reported by A. Chodera et al. (1991), which claimed a decrease in the excretion of these ions [9,65].

Besides the flavonoidic fraction, leiocarposide (25 mg/kg, i.p.) was also shown by A. Chodera et al. (1985) to have diuretic activity, equivalent to about 75% of the furosemide effect [9,130]. The same authors have demonstrated that the i.p. route results in higher efficacy (about 30%) than the oral route, that it has a slow onset (around 5 h), and it lasts for up to 24 h [9,119,131]. The aglycone part of the glycoside (leiocarpic acid) is devoid of diuretic activity (at the same dose, 25 mg/kg i.p.) [9,132].

Besides the assumed diuretic effects, S. virgaurea has been declared “the plant that is most frequently extracted to yield preparations for the treatment of bladder dysfunction including the overactive bladder syndrome” [31]. In this sense, clinical data in patients with dysuria have claimed that *S. virgaurea* reduces the frequency of urination (as well as the pain associated with it). In a relatively large (*n* = 512 patients), but open-label and uncontrolled study of patients with chronic recurrent overactive bladder, 96% of the subjects receiving 424.8 mg of *S. vigraurea* extract, t.i.d., reported an improvement in the clinical global impression and a significant drop in painful micturition and in the need to urinate [133]. In a smaller study (*n* = 74), also open-label, carried out on female patients with dysuria, a reduction of the same symptoms was observed in 69% of the patients [133]. These results are somewhat puzzling because diuretics tend to rather increase the frequency of urination, but the diuretic data come from non-clinical studies with an isolated compound (leiocarposide) or the flavonoid fraction, and not with an extract as such. On the other hand, the open-label character and the lack of a control group indicate a low quality of these data and stronger evidence is needed to conclude on the effect *S. virgaurea* have on the urinary tract. In vitro data have demonstrated that extracts of the plant have anti-muscarinic effects on the M2 and M3 receptors, which results in the inhibition of the bladder contraction [31].

Leiocarposide (25 mg/kg p.o.) administered for six weeks significantly inhibited the growth of human-derived urinary calculi transferred into the rat bladder [57].

### 6.6. Antibacterial Activity

An investigation of the antibacterial potential of two extracts, one alcoholic and one lipophilic (with hexane), found that the alcoholic had the lowest MIC for *Staphyllococcus aureus*, whereas the lipophilic one for *S. aureus* and *P. aeruginosa* [134]. The MIC values varied between 2.95 and 11.8 mg/mL for the ethanolic; for the hexane extract, MIC was 3.5 mg/mL for *Staphyllococcus aureus*, *Staphyllococcus faecalis*, and *Pseudomonas aeruginosa*, and higher than 3.5 mg/mL for the other microorganisms tested [134]. Although values lower than 16 mg/mL have been considered sometimes as showing a strong antibacterial effect [135], other authors have used more stringent criteria: MIC values < 100 μg/mL have been proposed to be highly active, those between 100 and 500 μg/mL active, those between 500 and 1000 μg/mL moderately active, those between 1000 and 200 μg/mL of low activity, and those with MIC > 2000 μg/mL inactive [136,137]. It has also been suggested that in order to “be considered a promising activity, a crude extract must demonstrate a MIC under 100 μg/mL” (and a pure compound less than 16 μg/mL) [138]. Other studies have also assessed various extracts for their antimicrobial effects, but MIC or MBC (minimal bactericidal concentration) was also for most species higher than 2000 μg/mL [139,140,141]. In the light of these criteria from the literature, the activities observed in this study are not “promising” and because they are higher than 2000 μg/mL, should rather be considered inactive.

In a study comparing (among others) the ethanol and aqueous extracts obtained from leaves and stems of *S. virgaurea* (agar disc diffusion method), inhibition zones were detected only for the ethanol extract and only on *Bacillus subtilis*, *Micrococcus flavus* (Gram-positive), and the Gram-negative *Morganella morganii* [142]. Another study also reported no inhibitory effect on several microbial species of an aqueous extract [72]. It has been suggested that inhibition zones smaller than 9 mm should be considered inactive, those ranging between 9 and 12 mm, moderately active, those ranging between 13 and 18 mm, active, and those larger than 18 mm should be classified as very active [136,143]. Since, in this study, all inhibition zones were less than 9 mm in size [142], the ethanol extract should also be regarded as virtually inactive. A. Brantner and J. Grein (1994), though, reported inhibition zones of around 11 mm on *Staphylococcus aureus* and *Escherichia coli* for an aqueous extract prepared from the aerial parts of the plant [140]. A methanol extract produced inhibition zones of 15 mm for *Staphylococcus aureus* and *Bacillus cereus*, and of 20 mm for *Enterobacter fecalis* [95] (i.e., the extract could be considered active and very active, respectively, on these microbial species).

A root extract was fractioned in a bioassay-guided manner and the active compounds responsible for the antibacterial effects (on *Bacillus subtilis* and *Xanthomonas euvesicatoria*, a plant pathogen) were identified as two isomers of the matricaria ester. For the extract, the MIC determined was 172 µg/mL for *B. subtilis*, and 86 µg/mL for *X. euvesicatoria* [32]. Nine clerodante-type diterpenes have been evaluated for their activity on *S. aureus*, and all had a modest activity, considerably inferior to that of vancomycin (the lowest MIC, observed for the second clerodane compound, was 30 µg/mL, 15 times higher than that of vancomycin and over the 16 µg/mL for a pure compound to be considered “active”) [78]. A. V. Tkachev et al. (2006) claimed complete inhibition of *S. aureus* for the essential oil [35], but these positive results were observed only with the undiluted essential oil and the ½ dilution, which is of very little clinical significance for systemic use.

Inhibition of bacterial cell division, impairment of plasmalema or cell membranes inside the cell resulting in cell lysis [134,144] and inhibition of dihydrofolate reductase [9,118] have been suggested as potential mechanisms for the supposed antibacterial effects of *S. virgaurea* extracts. The effect has been attributed to several phytochemical groups: phenolic acids and flavonoids [134], terpenes and essential oils [35,134], matricaria ester isomers [32], and clerodante-type diterpenes [78]. Considering the modest antibacterial activity reported by most studies, the discussion of these mechanisms (which are supported in a general way by the general literature on these phytochemicals [145,146,147,148]) is, in our view, rather moot.

### 6.7. Antifungal Activity

G. Bader et al. investigated the antifungal potential of *S. virgaurea* extracts and reported that deacylated saponins tend to have a stronger effect on different *Candida* species and *Cryptococcus neoformans* than the corresponding mixture of ester saponins, and among the deacylated saponins, the bidesmosides had stronger activity than monodesmosides [9,66,67,119,149]. *S. Pepeljnjak* et al. (1998) claimed antimycotic activity on several dermatophyte species (*Trichophyton mentagrophytes*, *Microsporum gypseum* and *Microsporum canis*) for a hydroalcoholic extract (75%) [150], but the MIC values (between 9.7% and 50%) seem too high to support the claim for clinical purposes, the effect being modest at best. M. Chevalier et al. (2012) reported no inhibitory effect of an aqueous extract from *S. virgaurea* on four strains of *Candida albicans* on agar mediums or in liquid medium, but they found that it was able to inhibit the transition between yeast and hyphal growth, a significant inhibition of biofilm formation, as well as a decrease of pre-formed biofilms of *C. albicans*, an effect speculatively attributed to the saponin fraction of the extract [72]. This attribution seems reasonable considering the inherent surfactant properties of saponins, as well as their hemolytic and iron chelator qualities, iron being necessary for the growth and development of *Candida* [72,151]. To confirm this, the same research group carried out a bioassay-guided fractionation that led to the isolation of six triterpene saponins (oleanane-type), of which four caused significant inhibition of *C. albicans* yeast-hyphal switch [73]. What is the clinical relevance of this effect only time will tell.

### 6.8. Antiparasite Activity

In a murine experiment, at a dose of 500 mg/kg bw, an extract of the aboveground parts of S. virgaurea showed the largest effect on *Acanthamoeba*, prolonging the survival of the animals to an average time of 12 days, whereas the controls had a mean survival time of only 4 days. Another three species tested in the same experiment were associated with shorter survival times [152]. An extract obtained from the stems did not have any remarkable activity on the parasitic nematode *Haemonchus contortus* [153].

### 6.9. Cytotoxic and Antitumor Activity

In murine models of sarcoma (allogenic sarcoma-180 and syngenic DBA/2-MC.SC-1 fibrosarcoma), significant antitumor activity was claimed for virgaureasaponin E administered in low doses (1 mg/kg/day) [9,68,69,154]. This effect of viragurea saponin E may at least partly be related to its ability to induce TNFα release from macrophages, as well as to an induction of cytotoxic macrophages [154]. Data for saponins from several species, including *S. virgaurea* L., evaluated on YAC-1 (T cell lymphoma) and P-815 cells (mouse mastocytoma), indicated that the glycosylation pattern (at 3 or 28 carbon atom position) has an influence on the cytotoxicity and that similarly to the antifungal effect, bisdesmosides derived from the polygalacic acid tend to be more active than their related prosapogenins [155]. Not only virgaureasaponin E is able to activate macrophages and TNF-α secretion, but also two C6-C1 compounds of the herbal product (2-methoxybenzyl-2-hydroxybenzoate, benzyl-2-hydroxy-6-methoxybenzoate) [116].

A methanol extract of *S. virgaurea* ssp. gigantean was reported to be active against the melanoma cell line SK-MEL-2 [50]. Fractionation guided by cytotoxicity led to the isolation of three active compounds from the hexane-soluble fraction, evaluated on five human tumor cell lines: erythrodiol-3-acetate, α-tocopherol quinone, and 2-phyten-l-ol [50]. However, the ED_50_ varied between 5.9 and 54.8 μM, which qualified the activity as rather modest (the most active one, with ED_50_ less than 10 μM on four out of the five cancer cell lines was the α-tocopherol quinone) [50]. It should also be considered, though, that if one important mechanism of antitumor activity is the stimulation of cytotoxic macrophages and TNFα release, as discussed above [154], a mere cytotoxicity test may not be appropriate to assess the antitumor potential of these extracts.

Experiments on PC3 cells have shown that, whereas aqueous and ethanol extracts had strong cytotoxic activity, chloroform extracts were devoid of such activity [34]. Extracts prepared from stems were markedly less active than those prepared from leaves or flowers [34]. The same authors also found that pre-treatment with protease leads to an almost complete loss of effect, which indicates that a protein is responsible for this activity [34]. A rodent model of prostate cancer (AT6.1) showed that i.p. administration in SCID mice of an active fraction corresponding to a molecular weight of about 40,000 Da (at a low dose of 5 mg/kg, once every three days) resulted in a significant reduction in tumor volume as compared with the non-treated animals [34]. Since SCID mice have immune deficiencies consisting of the lack of B and T lymphocytes, the authors speculated that the effect of this fraction is due to a direct cytotoxic effect rather than being mediated by the immune system [34]. This was also supported by the caspase-3 increase in cells treated with the fraction and the flow cytometric analysis, which suggested that the fraction causes cell cycle arrest (in the G0/G1 phase), triggering then apoptosis [34]. Since SCID mice, though, have macrophages, an immune contribution should not be necessarily excluded, in the absence of direct evidence to the contrary.

Ent-germacra-4(15),5,10(14)-trien-1β-ol, β–dictyopterol, and 3,5-di-*O*-caffeoyl quinic acid exhibited “moderate cytotoxicity” against five cancer cell lines, with ED_50_ values varying between 1.52–18.57 μM (the most active–ED_50_ between 1.52 and 18.57 μM–being β–dictyopterol) [49]. Methyl 3,5-dicaffeoyl quinate (MDQ) has demonstrated antioxidant activities, as well as antiproliferative effects in vitro, in HT-29 cell, by inducing cell cycle arrest and apoptosis through inhibition of the PI3K/Akt and ERK signaling pathways [156].

### 6.10. Antimutagenic Activity

Hexane extracts of three *Solidago* species demonstrated antimutagenic activity in a *S. typhimurium* test, the most active apparently being *S. virgaurea* L; instead, the ethanolic extracts were devoid of any such antimutagenic effects [134].

### 6.11. Antiadipogenic and Antidiabetic Activities

Following an impressive screening of about 300 plant extracts, kaempferol-3-*O*-rutinoside was isolated from a butanol fraction of a water extract of *S. virgaurea*, showing strong anti-adipogenic effects in vitro and supressing PPAR-γ and C/EBPα expression [100]. Of a number of several phenolic derivatives of the species, evaluated in vitro for their anti-adipogenic potential, 5-di-*O*-caffeoylquinic acid was found to have the most potent inhibitory effect [157]. A 10% ethanolic extract of *S. virgaurea* also showed good anti-adipogenic effects in vitro and in vivo in a murine model, causing a decrease in body weight, liver weight, and the magnitude of the adipose tissues [158]. Such experimental data are worth further investigation of the species for its potential use for weight loss purposes.

In an alloxan-induced diabetic rat model, a hydroalcoholic extract of *S. virgaurea* caused a decline in glycemia, TNF-α, serum amylase activity, and pancreatic malondialdehyde, as well as a rise in serum insulin, hepatic glycogen, pancreatic SOD, and catalase activities [159].

### 6.12. Cardioprotective Effects

In a rat model, a protective effect against cardiotoxicity (induced with isoproterenol) was claimed for a methanolic extract of *S. virgaurea* L. [160].

### 6.13. Antisenescence Effects

An alcoholic extract of *Solidago virgaurea* subsp. *alpestris* was claimed to exert anti-senescence effects in vitro on fibroblasts [161], opening a door towards the possibility to use it as for anti-aging purposes in topical or systemic preparations.

An overview of the pharmacological effects of extracts prepared from *S. virgaurea* L. is shown in Table 3.

## 7. Conclusions

*S. virgaurea* L. is a native species in Europe, with a long tradition of medicinal use for a variety of therapeutic purposes in different geographical reasons: urinary tract conditions, gastro-intestinal conditions, diabetes, allergies, as well as for healing and antiseptic purposes. The largest body of research has been limited to non-clinical experiments up to date, having a wide variability with respect to design, methodological quality, and evidence strength. Many of the relevant studies have been performed before 2000, published in other languages than English, and are not easily accessible today. Clinical investigation has been very limited in scope and volume, and the little clinical data available are not of the highest quality (no clinical randomized, controlled, double-blind study has been published thus far). Whereas some of the pharmacological activities have not been promising (e.g., the antibacterial, antifungal, or cytotoxic effects seem rather modest), others seem more interesting and invite to further research (e.g., antiadipogenic, antisenescence, or the beneficial effects in dysuria and overactive/irritable bladder).

## Figures and Tables

**Figure 3:**
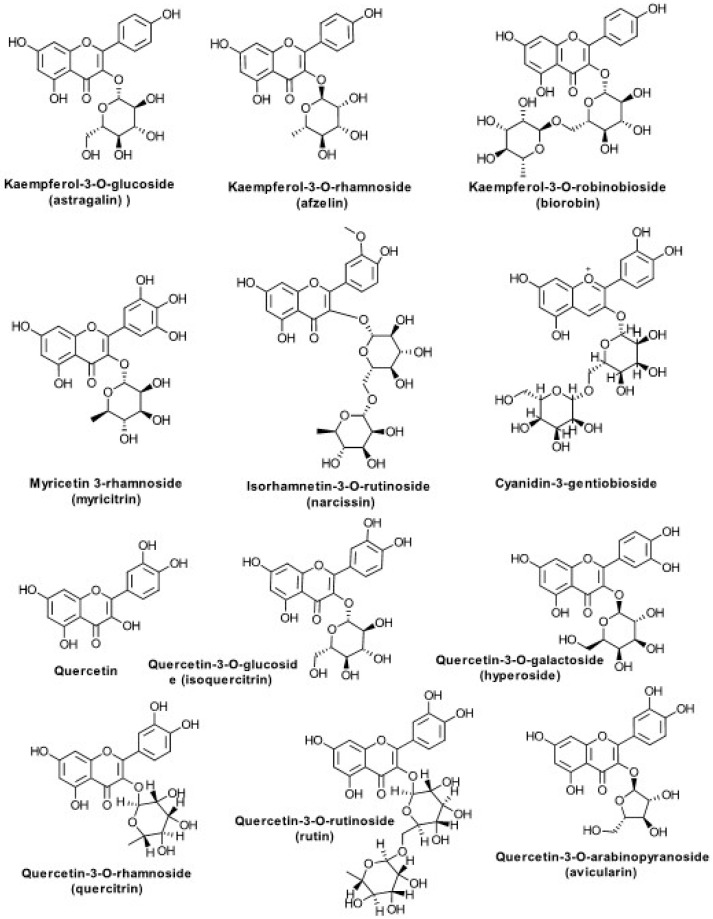
Representative flavonoids from *S. virgaurea* L.

**Figure 4:**
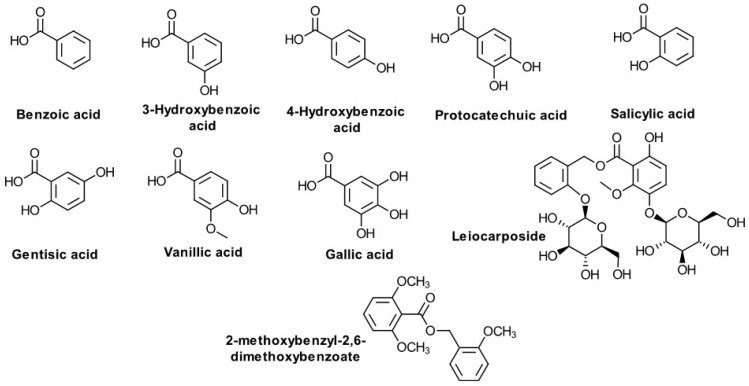
C6-C1 compounds from *S. virgaurea* L.

**Figure 5:**
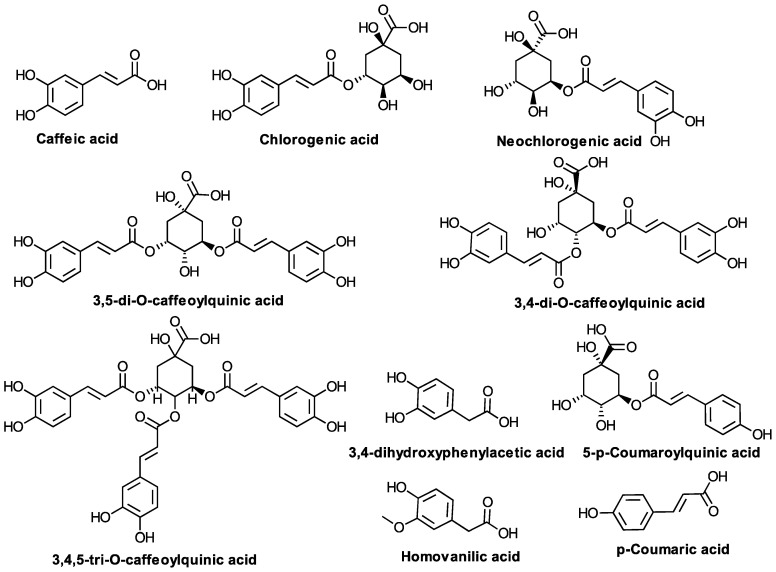
C6–C2 and C6–C3 compounds from *S. virgaurea* L.

**Figure 6:**
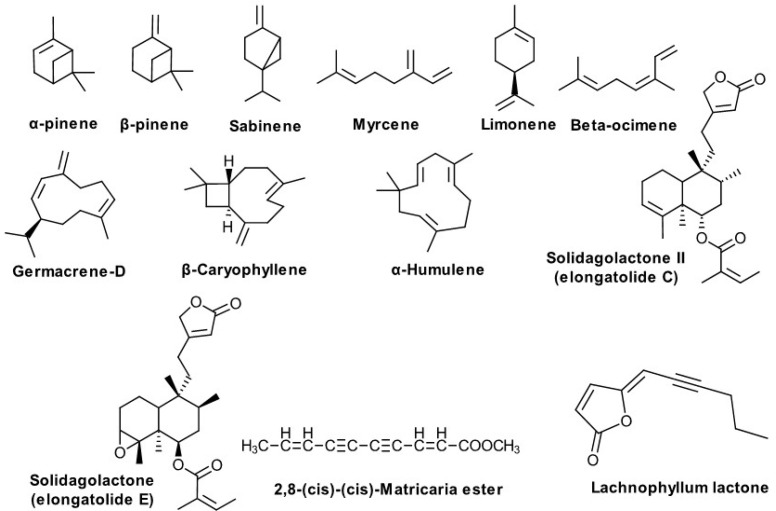
Terpenes from *S. virgaurea* L.

**Figure 7:**
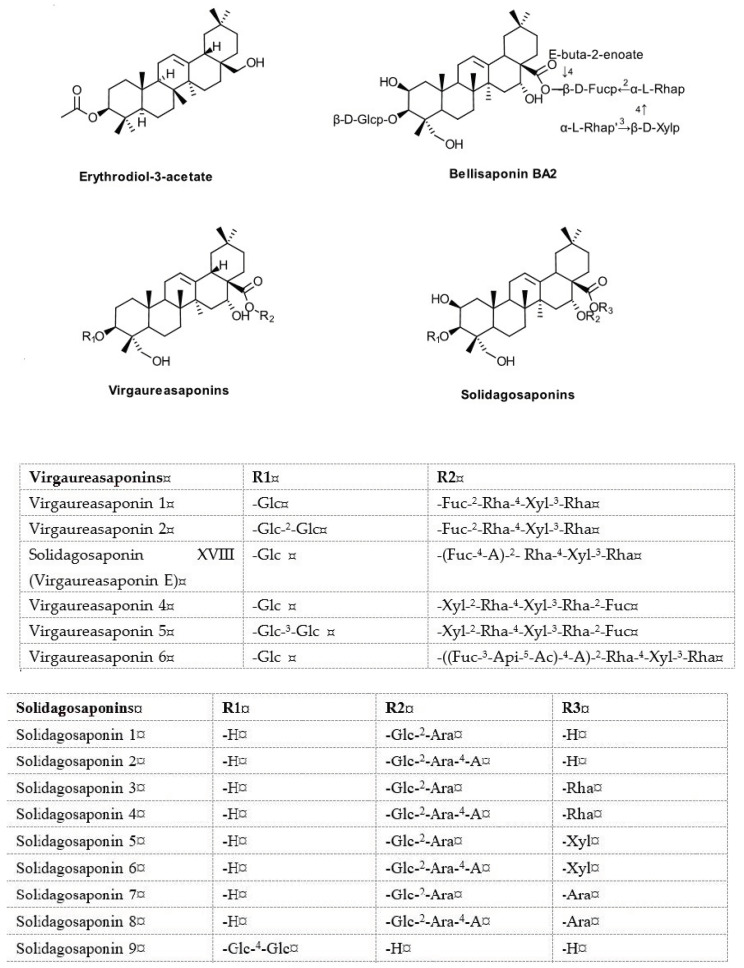
Saponins from *S. virgaurea* L.

**Table 2:** Detailed composition of the essential oil obtained from *S. virgaurea* L. flowering tops.

Compound	Proportion (%)	Reference(s)
α-Pinene	0.47–36.5	[35,76,77,78]
Camphene	0.02–0.6	[35,76,77,78]
Sabinene	0.06–11.8	[35,76,77,78]
Myrcene	0.05–17.9	[35,76,77,78]
β-Pinene	0.16–13.3	[35,76,77,78]
3-Carene	0.1–0.7	[35,76]
α-Terpinene	Tr. *–0.3	[76]
Limonene	0.07–14.8	[35,76,77,78]
*p*-Cymene	Tr. *–0.77	[76,77,78]
(E)-β-Ocimene	0.02–4.7	[35,76,78,79]
Linalol	0.3–0.8	[76]
Nonanal	Tr. **–1.4	[76,77]
trans-Verbenol	Tr. *–0.7	[76,77]
trans-Pinocarveol	0.09–0.2	[76,77]
Decanal	0.04–0.7	[35,76,77]
Terpinen-4-ol	0.1–1.1	[76,77,78]
Borneol	Tr. *	[76,77]
α-Terpineol	0.13–1.89	[76,77,78]
γ-Terpineol	0.04–0.2	[76]
*p*-Cymen-8-ol	0.03–0.51	[76,78]
*trans*-Carveol	0.05–0.3	[76,77]
Myrtenal	Tr. *–0.06	[76,77]
Geraniol	0.02–0.45	[76,78]
Verbenone	Tr. *–0.6	[76,77]
α-Cubebene	Tr.*–2.35	[76,77,78]
δ-Elemene	Tr. *–9.38	[35,76,77,78]
Bornyl acetate	0.13–4.52	[35,76,77,78]
Carvone	Tr. *–0.4	[76,77]
α-Copaene	Tr. *–0.64	[35,76,77,78]
β-Bourbonene	0.2–7.28 *	[76,77,78]
β-Cubebene		[76,77,78]
β-Elemene		[35,76,77,78]
Geranyl acetate	Tr. *–0.2	[35,76]
Isobutyl benzoate	Tr. *	[76]
(Z)-β-Farnesene	Tr. *–0.6	[76,77,78]
β-Caryophyllene	0.1–10.5	[35,76,77,78]
α-Humulene	0.1–4.1	[35,76,77,78]
γ-Muurolene	Tr. *–1.86	[35,76,77,78]
Germacrene-D	0.1–17.68	[35,76,77,78]
Isoamyl benzoate	0.08–0.4	[35,76]
α-Muurolene	Tr. *–3.6	[35,76,77,78]
Bicyclogermacrene	Tr. *–0.9	[35,76,77]
γ-Cadinene	Tr. *–0.7	[35,76]
Nerolidol	0.07–0.6	[35,76,77,78]
Calamenene A	Tr. *–0.2	[76,77,78]
Caryophyllene epoxide	0.4–1.6	[76,77]
Spathulenol	0.29–11.33	[76,78]
(Z)-hex-3-enyl benzoate	0.08–0.8	[76,77]
Torreyol	Tr. *–0.6	[76,77]
T-Muurolol	0.2–1.16	[76,77]
Humulene epoxide	0.2–0.5	[76,77]
α-Cadinol	Tr. **–3.06	[35,76,77,78]
(Z)-hex-3-enyl salicylate	0.09–0.3	[76,77]
Eudesma-4(15),7-dien	0.1–0.2	[76]
Mintsulphide	Tr. *	[76]
Cyclocolorenone	Tr. *–0.3	[76,77]
Benzyl benzoate	Tr. *–57.0	[35,76,77,78]
Geranyl benzoate	Tr. *–0.1	[76]
Benzyl salicylate	0.02–1.14	[35,76,77,78]
β-Phenylethyl salicylate	0.1–0.6	[76,77]
α-Thujene	Tr. *	[77]
Linalool	0.23–2.0	[77,78]
Perillene	0.3	[77]
Campholene aldehyde	0.6	[77]
Pinocarvone	0.5	[77]
*ar*-Curcumene	0.5	[77]
Spathulenol	1.6	[77]
Salvial-4(14)-en-1-one	0.1	[77]
Torilenol	0.8	[77]
Junenol	0.2	[77]
Eudesma-4(15),7-dien-1β-ol	0.1	[77]
Acetone	0.02–0.62	[78]
Ethyl acetate	0.02–2.27	[78]
Ethyl alcohol	0.03–1.62	[78]
α-Phellandren	Tr. *–1.12	[78]
β-Phellandren	0.07–0.26 ***	[35,78]
Terpinolene	Tr. *–0.2	[35,78]
*n*-Hexanol	0.02–0.10	[78]
*cis*-3-hexen-1-ol	0.16–1.52	[78]
*trans*-Linalool-oxide	Tr. *–0.11	[78]
*trans*-sabinen-hydrate	Tr. *–0.26	[78]
*cis*-Linalool-oxide	Tr. *–0.06	[78]
Benzaldehyde	0.13–0.40	[78]
γ-Elemene	Tr.*–0.06	[78]
Aromadendrene	0.03–0.20	[78]
Acetophenone	Tr. *–0.19	[78]
Salicyl aldehide	Tr. *–0.02	[78]
*trans*-β-Farnesene	0.07–4.80	[35,78]
Germacrene-B	0.03–16.63	[78]
*cis*-α-Farnesene	Tr. *–0.41	[78]
*δ*-Cadinene	0.2–7.87	[35,78]
Cubenene	0.02–0.25	[78]
Benzyl alcohol	0.19–1.80	[78]
2-Phenyl ethyl alcohol	0.09–1.18	[78]
*o*-Methoxy benzaldehyde	0.02–0.38	[78]
Caryophyllene oxide	0.1–4.30	[35,78]
Epi-cubenol	0.04–2.79	[78]
*o*-Methoxy benzyl alcohol	0.07–1.29	[78]
T-Cadinol	0.12–0.76	[78]
T-Muurolol	0.25–1.16	[78]
δ-Cadinol	0.15–0.73	[78]
*n*-Tetracosane	Tr. *–0.24	[78]
*n*-Pentacosane	Tr. *–0.50	[78]
Phytol	0.02–1.07	[78]
n-hexacosane	Tr. *–0.30	[78]
Myristic acid	Tr. *–1.30	[78]
β-Ocimene-Y/(Z)- β-ocimene	0.02–3.0	[35,78,79]
γ-Terpinene	Tr. **	[35]
1-undecene	Tr. **–0.1	[35]
4,8-dimethyl-1,3,7-nonatriene	0.1	[35]
Camphor	Tr. **–0.2	[35]
Zingiberene	0.4–1.1	[35]
Germacrene A	0.1–0.7	[35]
(E,E)- α -farnesene	1.0–2.7	[35]
β-sesquiphellandrene	0.1–0.2	[35]
(Z)-3-hexenyl benzoate	0.1–0.4	[35]
β-Eudesmol	Tr. **–0.1	[35]
Neophytadiene	0.1–0.2	[35]
2-phenylethyl benzoate	Tr. **–0.4	[35]

* Tr.—traces (<0.02%); ** Tr.—traces (<0.1%) (different cut-off levels were used in different papers to define “traces”). *** “limonene + β -phellandrene (2:1)”: 1.8–6.4% [35].

**Table 3:** A summary of the pharmacological properties of extracts and fractions obtained from *S. virgaurea* L.

Pharmacological Properties	Evidence	Chemical Compounds/Fraction to Which the Property is Ascribed	Critical Assessment of Study Results
Antioxidant effects	In vitro	Caffeoylquinic acids	Moderate antioxidant effects (11th position among 23 herbal products)
Antiinflammatory effects	In vitro and in vivo (at least 4 rat studies)	Triterpenes, leiocarposide, rutin and quercetin, caffeoylquinic derivatives	Effect not superior to conventional NSAIDs (phenylbutazone, indomethacin)
Analgesic activity	In vivo (one study in mice)	Leiocarposide	Similar effects to aminophenazone, but very short duration (one hour)
Spasmolytic activity	In vitro or ex vivo	NA	Modest spasmolytic effect (less than 15% of the papaverine effect)
Antihypertensive activity	*In vivo* (one study in dogs and one in rats)	Antihypertensive activity attributed to flavonols	Contradictory results on blood pressure
Diuretic effects	In vitro, in vivo (at least three rat studies)	Flavonoid fraction (quercetin and its derivatives), hydroxycinnamic acid fraction, saponin fraction, leiocarposide	Effects comparable to those of furosemide or slightly inferior for several fractions
Control of overactive bladder symptoms	In vitro data showing antimuscarinic effects and two clinical, open, un-controlled data	NA	Low quality, but promising data. Contradictory findings between reduction of urination frequency seen in clinical data and diuretic effects reported in non-clinical studies
Antilithiatic effects	One rat study	Leiocarposide	Additional confirmation needed
Antibacterial effects	In vitro data only	Matricaria ester isomers, clerodane diterpenes (but their effects are rather modest)	Modest effects in the majority of studies. One disc diffusion study reported encouraging results on *S. aureus* and *E. fecalis* for a methanol extract.
Antifungal activity	In vitro data only	Triterpene saponins	Modest effects for most fungi tested up to date. Inhibition of inhibition of *Candida* biofilm formation and of yeast-hyphal switch seems more promising, but the clinical relevance is unclear.
Antiparasite activity	One in vivo study for *Acanthamoeba,* one in vitro study for *Haemonchus contortus*	NA	Promising results for *Acanthamoeba* (additional confirmation needed). No effect against *H. contortus*
Cytotoxic and antitumor activity	Mostly in vitro data, one in vivo study (mice)	Saponins, α-tocopherol quinone, 2-phyten-l-ol, a protein, β–dictyopterol, methyl 3,5-dicaffeoyl quinate	Moderate effects against some cancer cell lines. More research needed.
Antimutagenic activity	In vitro (one study)	Hexane soluble fraction	Effects observed at a quite high concentration (2.5 mg/mL).
Antiadipogenic effects	In vitro (two studies) and in vivo (one murine study)	5-di-*O*-caffeoylquinic acid	Promising results, further data necessary
Antidiabetic effects	One rat model	Hydro-alcoholic fraction	More data necessary
Cardioprotective effects	One rat model	NA	More data necessary
Antisenescence effects	One in vitro study	NA	More data necessary
